# The inhibitory effect of a fermented papaya preparation on growth, hydrophobicity, and acid production of *Streptococcus mutans*, *Streptococcus mitis*, and *Lactobacillus acidophilus*: its implications in oral health improvement of diabetics

**DOI:** 10.1002/fsn3.55

**Published:** 2013-09-30

**Authors:** Jhoti Somanah, Emmanuel Bourdon, Theeshan Bahorun, Okezie I Aruoma

**Affiliations:** 1 ANDI Center for Biomedical and Biomaterials Research, University of MauritiusRéduit, MSIRI Building, Mauritius, Republic of Mauritius; 2 Groupe d'Etude sur l'Inflammation Chronique et l'Obésité, Université de La RéunionPlateforme CYROI, Saint Denis, France; 3 School of Pharmacy, American University of Health SciencesSignal Hill, California 90755, USA

**Keywords:** Antimicrobial activity, dental caries, diabetics, fermented papaya preparation, gingivitis, oral health and hygiene, oral microbial infections

## Abstract

Fermented papaya preparation (FPP) is a “natural health product.” The high incidence of dental caries, gingivitis, periodontitis, and oral microbial infection cases among patients with diabetes mellitus continues to prevail. The potential role of FPP against common oral microbiota (*Streptococcus mutans*, *Streptococcus mitis*, and *Lactobacillus acidophilus*) isolated from the human oral cavity was investigated using in vitro simulation models of dental plaque and caries. FPP showed an inhibitory effect against the growth (at 0.05 mg/mL: *S. mutans*: −6.9%; *S. mitis*: −4.47%, *P* < 0.05), acid production (at 0.05 mg/mL: *S. mutans*: +6.38%; *L. acidophilus*: +2.25%), and hydrophobicity (at 50 mg/mL: *S. mutans*: 1.01%, *P* < 0.01; *S. mitis*: 7.66%, *P* < 0.05) of tested microbiota. The results of this study suggest that low doses of FPP may be a suitable complement to good oral hygiene practice for the effective prevention of dental caries, plaque, and gingivitis. The functional application of FPP as a constituent of a balanced diet and active lifestyle can make a positive contribution to the oral health status and well-being of patients with diabetes.

## Introduction

Epidemiological surveys have brought to light the high prevalence of dental caries, gingivitis, periodontitis, and oral microbial infection cases among patients with diabetes mellitus. In an attempt to explain this trend, multiple pathophysiological mechanisms have been suggested, including deficient nutritional intake, alterations in host response to oral microflora, compromised neutrophil function, and decreased phagocytosis and leukotaxis. Bacteria-triggered secretion of serum pro-inflammatory cytokines in the mouth may induce hyperglycemia and ultimately cause insulin resistance and contribute to the indirect destruction of pancreatic beta cells (Tsai et al. 2002). A realistic management plan that includes regular oral hygiene practice and basic dental treatment is therefore fundamental for managing diabetes and its associated oral complications. Nowadays, active constituents extracted from plants are often included in the preparation of toothpaste, mouth rinses, dental floss, and chewing gum to ensure a stronger antimicrobial activity (Bone 2005). Ongoing studies focusing on the anticariogenic properties of polyphenols isolated from green tea (Otake et al. 1991), red chicory (Canesi et al. 2011), cranberry juice (Babu et al. 2012), and shiitake mushrooms (Signoretto et al. 2011) look promising. However, despite the numerous studies conducted on such functional foods, only a handful of plants can be clinically used to control dental plaque, caries formation, and mouth infections due to their effectiveness, stability, taste, and economic feasibility (Bagramian et al. 2009). Interestingly, dietary agents often lack bactericidal activity after commercial production, but retain their ability to manipulate oral microbiota by exhibiting other important properties such as antiadhesion, antibiofilm, and anti-inflammatory. Fermented papaya preparation (FPP: also known as Immun'Age®; Osato Research Institute, Gifu, Japan) is one such functional food. Clinical data now support the use of FPP as a dietary supplement in the management of type 2 diabetes mellitus by virtue of its ability to effectively reduce fasting blood glucose levels, low-density lipoprotein/high-density lipoprotein ratio, and inflammatory biomarkers such as C-reactive protein and uric acid (Danese et al. 2006; Somanah et al. 2012). In this context, FPP may prove to be a valuable asset in reducing the risk of developing oral pathologies such as dental caries and gingivitis. Collard and Roy (2010) reported a reduction in inflammation of the gums in FPP-supplemented rats with gingivitis. Despite postulations of the involvement of β-d-glucans (the major structural constituent of yeast cell walls) and complex amino acid and carbohydrates, the mechanisms behind the immunomodulatory role of FPP are still to be clarified (Islam et al. 2008). The current lack of comparative data of FPP in this particular domain of oral health has prompted us to carry out a preliminary assessment of the antimicrobial activities of FPP. Further studies are warranted to provide a comprehensive insight into the molecular inhibitory mechanisms demonstrated by FPP in this study. Indeed, oral health in communities affected by diabetes continues to impact health expenditures on dental services.

## Methodology

Prior to conducting each assay, American Type Culture Collection (ATCC) strains of *Streptococcus mutans* (25175), *Streptococcus mitis* (6249), and *Lactobacillus acidophilus* (4356) were grown for 24 h to reach stationary phase in sterile brain heart infusion (BHI: for *Streptococcus* strains) or MRS broth (for *L. acidophilus*) at 37°C. Standardization of bacterial suspension was made using sterile 0.85% sodium chloride with comparison against McFarland standards.

### Effect of FPP against the growth of oral microbes

Using the modified method of Islam et al. 2008, an aliquot of 50 μL standardized microbial suspension (2 × 10^8^ CFU/mL) was added to 3 mL fresh BHI or de Man, Rogosa and Sharpe (MRS) broth, 300 μL FPP (Osato Research Institute, Gifu, Japan, 0.05–50 mg/mL), and 100 μL tween 80 (0.001% v/v). Ciprofloxacin was used as positive control. All cultures were incubated for 24 h at 37°C. At the end of the incubation period, turbidometric analysis was performed at 600 nm against a blank of plain broth.

### Effect of FPP on the adherence of oral microbes to a glass surface

The glass surface adherence assay was performed according to the modified method of Islam et al. 2008). To 3 mL BHI or MRS broth, 50 μL standardized microbial suspension (2 × 10^8^ CFU/mL), 300 μL FPP (0.05–50 mg/mL), 300 μL sucrose (or glucose for *L. acidophilus*) (1%), and 100 μL tween 80 (0.001%) were added. Ciprofloxacin was used as positive control. All tubes were inclined at an angle of 30° and incubated at 37°C for 24 h. After overnight incubation, the supernatant was decanted into a clean tube and adhered cells were removed by the addition of 3 mL sodium hydroxide (0.5 M). Both test tubes were centrifuged (1048 g, 15 min) and the aqueous supernatant discarded. Bacterial cells were resuspended in 3 mL sodium hydroxide (0.5 M) and the percentage of adherence was quantified at 600 nm by applying the following formula (Islam et al. 2008):


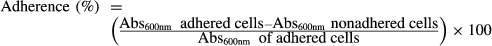


### Effect of FPP on the hydrophobicity of oral microbes

The cell surface hydrophobicity was measured according to the modified method of Ooshima et al. (2000). All bacterial strains were grown in 50 mL BHI or MRS broth for 24 h at 37°C. After incubation, cells were harvested by centrifugation (1048 *g*, 15 min), washed, and suspended in phosphate-urea-magnesium buffer (pH 7.1) to an optical density of 0.5–0.6 at 600 nm. To 1 mL of bacterial suspension, an equal volume of FPP (0.05–50 mg/mL) was added. After standing at room temperature for 30 min, 300 μL of *n-*hexadecane was added and agitated uniformly on a vortex for 1 min. After allowing complete separation of the aqueous phases, the upper phase was discarded and the cell density of the lower aqueous phase was determined at 600 nm. The percentage of cells partitioned to the hexadecane was calculated as the loss in absorbance relative to that of the initial absorbance using the following formula (Ooshima et al. 2000):


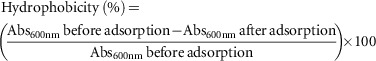


### Effect of FPP on acid production of oral microbes

Using the modified method of Ooshima et al. (2000), 20 mL phenol red broth containing 3% glucose (w/v) was inoculated with 300 μL standardized microbial suspension (2 × 10^8^ CFU/mL) followed by the addition of 1 mL FPP (0.05–50 mg/mL). Using a calibrated pH meter, the pH of the culture media was recorded before and after an incubation period of 24 h (for *Streptococcus* strains) or 48 h (for *L. acidophilus*) at 37°C.

### Statistical analysis

The results presented are expressed as mean values of triplicate experiments, where error bars represent ± standard deviation. Both descriptive and inferential statistical analyses were carried out using Microsoft® Office Excel® 2007 (version 12.0.6662.5003). Significant testing was performed by Student's paired *t-*test by comparing the mean values of two samples. For data which followed a nonnormal distribution, the nonparametric alternative Wilcoxon test was used. Differences were considered significant for a value of *P* < 0.05 (two tailed).

## Results and Discussion

**Figure 1:**
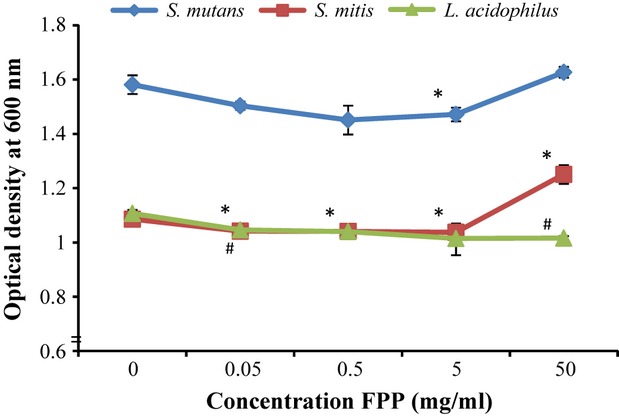
Effect of fermented papaya preparation (FPP) on the growth of *Streptococcus mutans*, *Streptococcus mitis*, and *Lactobacillus acidophilus*. Values are expressed as mean optical density at 600 nm of triplicate determinations (*n* = 3), where error bars represent ± standard deviation. Significance: *^/#^*P* < 0.05 versus control.

Figure 1 illustrates the dose-dependent effect of a FPP on the growth of *Streptococcus* and *L. acidophilus* after a 24-h incubation period. For all three strains, the inhibitory effect of FPP was observed between the concentration ranges 0.05 and 5 mg/mL. *S. mitis* and *L. acidophilus* were found to be more significantly susceptible to FPP. As the carbohydrate content is considerably elevated (∼0.97 g per g^−1^ FPP) (Aruoma et al. 2010), dextrose may have negatively influenced bacterial growth through osmotic dehydration of cells. Engels et al. (2009) reported an inhibitory effect of dried unripe pulp of *Mangifera indica* (mango) against *Bacillus* spp., *Pseudomonas* spp., and *S. mutans*. Interestingly, gallotannins purified from *M. indica*, although reported to have no effect on lactic acid bacteria, negatively influenced the proliferation of gram-positive bacteria through its metal-chelating ability. Indeed FPP may have the ability to chelate iron ions from the broth medium and deprive bacteria from normal growth (Prus and Fibach 2012). However, the growth increase at 50 mg/mL for *S. mutans* and *S. mitis* deserves further investigation as to the potential mechanisms involved.

**Figure 2:**
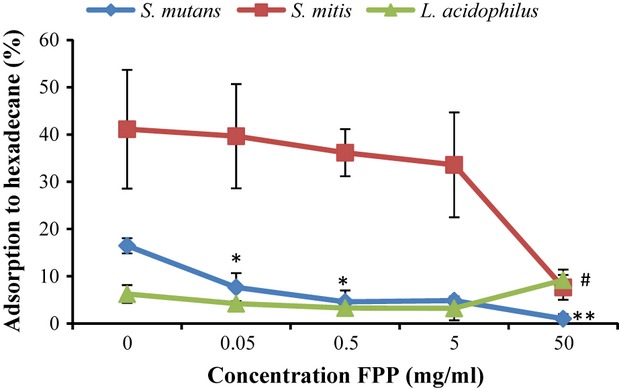
The effect of fermented papaya preparation (FPP) on cell surface hydrophobicities of *Streptococcus mutans*, *Streptococcus mitis*, and *Lactobacillus acidophilus*. Values are expressed as mean (%) of triplicate determinations (*n* = 3), where error bars represent standard deviation. Significance: *^/#^*P* < 0.05, **P < 0.01 versus control.

Through the use of hexadecane and the glass surface mimicking the hydrophobic nature of teeth or gum surface, it was found that FPP could significantly reduce bacterial affinity to hexadecane (Fig. 2) and biofilm formation independent of sucrose (at 0.05 mg/mL, Table 1) in a concentration-dependent manner. Melanoidin isolated from barley coffee, a beverage made from roasted barley, demonstrated an antiadhesive effect on *S. mutans* (Papetti et al. 2007). Interestingly, melanoidin remains undetected in unroasted barley, indicating that it forms uniquely upon fermentation (Papetti et al. 2007). Similarly, black tea potently reduced caries formation in hamsters by 63.7% (*P* < 0.05; fed a cariogenic diet) and by 56.6% (*P* < 0.05; fed a normal diet), in comparison with oolong tea extract (Linke and LeGeros 2003). Such findings imply that the extent of fermentation can influence its anticariogenic activity in vivo to exceed that of nonfermented extracts. There is a positive association between daily consumption of fermented papaya and antioxidant enzyme expression and their activities in vivo (Aruoma et al. 2006; Marotta et al. 2010). Compared to fresh papaya extracts, the enhanced dietary composition of FPP has resulted in the formation of novel amino acids and carbohydrates that are polyphenolic in nature and may interact with major bacterial hydrophobins.

**Table 1:** The effect of a fermented papaya preparation (FPP) on adherence of *Streptococcus mitis*, *Streptococcus mutans*, and *Lactobacillus acidophilus* to a glass surface in the presence or absence of 1% sucrose/glucose

		Concentration of FPP (mg/mL)				
		
Cell adherence (%)		0	0.05	0.5	5	50
*Streptococcus mutans*	1% sucrose	50.33 ± 3.62	43.79 ± 4.44	46.00 ± 3.11	47.57 ± 1.70	53.85 ± 0.49
	No sucrose	47.21 ± 2.24	37.07 ± 6.91	32.32 ± 4.48^*^	31.59 ± 4.63	35.51 ± 5.21
*Streptococcus mitis*	1% sucrose	76.00 ± 2.20	70.08 ± 7.12	72.86 ± 3.13	76.07 ± 3.29	77.16 ± 2.61
	No sucrose	62.52 ± 6.07	59.65 ± 1.97	63.76 ± 3.07	66.48 ± 2.87	68.38 ± 3.51
*Lactobacillus acidophilus*	1% glucose	82.14 ± 5.71	64.34 ± 4.50	68.75 ± 8.50	88.53 ± 5.51	93.60 ± 3.98
	No glucose	84.78 ± 11.7	84.06 ± 2.78	85.41 ± 9.77	85.82 ± 4.04	92.40 ± 2.08

Values are expressed as mean (%) ± standard deviation of triplicate determinations (*n* = 3).

Significance: ^*^P < 0.05 versus control.

**Figure 3:**
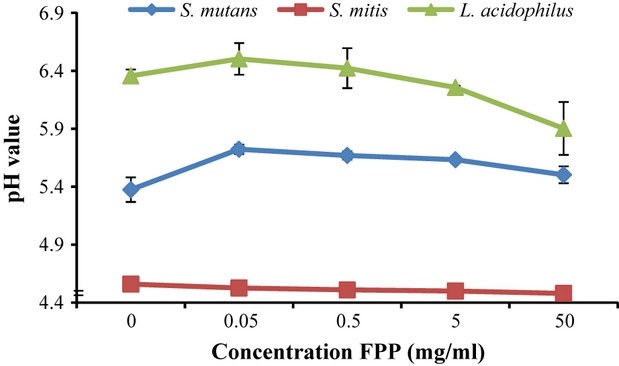
Effect of fermented papaya preparation (FPP) on the acid production of *Streptococcus mutans*, *Streptococcus mitis*, and *Lactobacillus acidophilus*. Values are expressed as mean pH values recorded after a 24-h incubation of triplicate determinations (*n* = 3), where error bars represent ± standard deviation.

The acidification of the basal medium in response to increasing concentrations of FPP was addressed in the course reported. Final pH reached was 5.50, 4.48, and 5.90 for *S. mutans*, *S. mitis*, and *L. acidophilus*, respectively. Fermentable carbohydrates are present in a sufficient amount in FPP to provoke a rapid drop in the pH level (Fig. 3). Despite elevated quantities of various amino acids in FPP, such as arginine, leucine, glutamic acid, and aspartic acid, the buffering effect was too small to counteract the quantity of acid being produced by the bacteria. Such amino acids have been reported to be utilized as a source of nitrogenous compounds to support bacterial growth (Hamid et al. 1994; Takahashi and Yamada 2000). For instance, *Porphyromonas gingivalis* and *Prevotella intermedia* (two common anaerobic colonizers present in gingival crevices) are able to produce acetic and succinic acids from aspartic acid (Takahashi and Yamada 2000). Similarly, valine and leucine can be degraded to isobutyric acid, iosvaleric acid, or isocarpic acid, respectively, by *Eubacterium* spp. (Hamid et al. 1994). The present findings of this assay provide additional evidence to support the results obtained for the growth and nonsucrose-dependent glass adherence assay (Fig. 1 and Table 1). The pH of oral plaque can be influenced by factors such as the saliva flow rate and oral clearance of nutrients from the mouth. Despite that the results of this study indicate that high concentrations of FPP may be acidogenic and have low bacterial tolerance, it is opined that given the refined powdery consistence of FPP and the fact that it is highly soluble in water, upon its oral consumption, it would be capable of stimulating secretion of an appropriate quantity of saliva and therefore FPP would rapidly clear from the oral cavity and thus encourage a rapid return to baseline (normal) pH.

The studies reviewed by Aruoma et al. (2006) have alluded to the potential of FPP to modulate oxidative injury as well as injury due to inflammation and improve the immune function. This supports the context that functional nutraceuticals and/or food supplements (as exemplified with FPP) exhibiting anti-inflammatory, antioxidant, immunostimulatory (at the level of the mucus membrane), and induction of antioxidant enzymes may have beneficial prophylactic potential in the management of chronic diseases characterized by oxidative stress and overt inflammation. That the antimicrobial activity of FPP could be boosted in synergy with other fermented plant-based foods is an interesting area for further investigation.

## Conclusion

Emerging data from this study are highly suggestive that low doses of FPP can be considered as a therapeutic functional food and can indeed counteract the action of various oral microbiota. *Carica papaya* contains many biologically active compounds such as alkaloids, flavonoids, glucosides, and anthraquinones, whose levels vary among its fruit, leaves, roots, and latex, and can modulate the proliferation of common bacteria and fungi. Despite the obvious differences between the composition and activity of fresh and FPPs, few deductions could be made from this study: the biofermentation process involved during its manufacture is likely to have contributed to its highly complex amino acid and carbohydrate profile. Modification of such molecules has generated “building blocks” that have been utilized to support the proliferation and growth of bacteria. At the same time, these modified molecules are suspected to interact with bacterial hydrophobins. It is clear that a study to explore the potential mechanism of action involved is warranted in the future. This study provides preliminary evidence of a determinant role of FPP in the protection against the risk of oral pathologies commonly linked to hyperinsulinemia, hyperglycemia, oxidative stress, and inflammatory states experienced during type 2 diabetes mellitus.
